# Animal Models Used in Monkeypox Research

**DOI:** 10.3390/microorganisms10112192

**Published:** 2022-11-04

**Authors:** Marianna Domán, Enikő Fehér, Renáta Varga-Kugler, Ferenc Jakab, Krisztián Bányai

**Affiliations:** 1 Veterinary Medical Research Institute, H-1143 Budapest, Hungary; 2 National Laboratory of Virology, Virological Research Group, Szentágothai Research Centre, University of Pécs, H-7624 Pécs, Hungary; 3 Department of Pharmacology and Toxicology, University of Veterinary Medicine, H-1078 Budapest, Hungary

**Keywords:** animal models, emerging infectious diseases, monkeypox virus, reservoir, vaccine, zoonosis

## Abstract

Monkeypox is an emerging zoonotic disease with a growing prevalence outside of its endemic area, posing a significant threat to public health. Despite the epidemiological and field investigations of monkeypox, little is known about its maintenance in natural reservoirs, biological implications or disease management. African rodents are considered possible reservoirs, although many mammalian species have been naturally infected with the monkeypox virus (MPXV). The involvement of domestic livestock and pets in spillover events cannot be ruled out, which may facilitate secondary virus transmission to humans. Investigation of MPXV infection in putative reservoir species and non-human primates experimentally uncovered novel findings relevant to the course of pathogenesis, virulence factors and transmission of MPXV that provided valuable information for designing appropriate prevention measures and effective vaccines.

## 1. Introduction

The emergence of the human monkeypox virus (MPXV), recorded now in 106 countries worldwide, has attracted massive attention from health experts, scientists and policymakers (data received by WHO national authorities until 3 October 2022) (https://www.who.int/publications/m/item/multi-country-outbreak-of-monkeypox--external-situation-report. Accessed between 7–5 October 2022). Understanding the epidemiology and infection biology of this neglected zoonotic pathogen has become a priority. MPXV is currently the most important orthopoxvirus affecting humans, probably as a result of waning herd immunity after the cessation of routine smallpox vaccination over four decades ago [1]. Based on the genomic sequence, MPXV strains may be phylogenetically separated into the West African (WA) and the Central African or Congo Basin (CB) clades, and the latter clade is divided into five groups. Unlike the WA clade, the cases associated with the CB clade have been connected predominantly to human-to-human transmissions with more severe clinical symptoms. WA-related monkeypox cases are characterized by a lower mortality rate and less serious illness [2,3]. Thus far, all reported human monkeypox infections reported from areas other than Africa have been caused by the WA strains, while CB MPXVs have primarily affected countries in Central Africa [1,4,5].

The clinical presentation of human zoonotic monkeypox includes disease progression through an incubation period, a pre-eruptive stage and a rash similar to smallpox. Lymphadenopathy is seen in up to 90% of patients during the pre-eruptive stage, which appears to be a prominent feature of monkeypox [6]. MPXV infection is characterized by an incubation period of 12–14 days in humans followed by a prodrome of fever for 1–3 days and nonspecific symptoms including chills, headaches, lethargy, asthenia, lymph node swellings, back pain and myalgia. Disseminated skin lesions begin to appear first as macules, then develop into papules, vesicles and pustules within 1–5 days after the onset of fever, first on the face, then across the body, hands, legs and feet. Scabs appear approximately 4 weeks after the initial infection [6,7].

The transmission of this zoonotic disease to humans could occur by direct contact with blood, body fluids or through mucocutaneous lesions of an infected animal. Interhuman and nosocomial transmission via respiratory droplets and contact with body fluids, contaminated environments and skin lesions of an infected person has also been increasingly reported [8,9,10,11]. In addition, MPXV transmission by sexual contact has been recently demonstrated to play a role in maintaining outbreaks [12,13]. To elucidate the infection biology of MPXV with regard to the cellular and viral factors that regulate virus transmissibility, infection and its maintenance in nature, studies with potential reservoir species have been carried out.

Serological and genetic evidence of MPXV infection suggests that a wide variety of animal hosts, such as rodents and perhaps primates, may be responsible for the circulation of MPXV in its native range, but ecological aspects of MPXV maintenance are still poorly understood. Identification of the true spectrum of animal reservoirs may be particularly useful to extend our knowledge about the transmission of MPXV and disease progression and promote the development of vaccines and antivirals. In addition, understanding viral shedding in potential natural hosts provides substantial information required to assess the risks for humans in contact with these species both around human settlements and natural habitats. Animal models of MPXV infection revealed similar route(s) of transmission and pathology as observed in humans [14,15]. Several MPXV-infected animal models have been established, including non-human primates (NHPs) (e.g., *Macaca fascicularis* and *M. mulatta*) and wild rodents (e.g., *Funisciurus anerythrus* and *Cynomys ludovicianus*) that could be utilized to elucidate the progress of MPXV infection and evaluate the efficacy of new generation non-replicating smallpox vaccines and recently developed antivirals against monkeypox. The primary objective of the present review is to summarize the current knowledge about animal infection of MPXV, including information acquired from cases of natural, accidental and experimental infections of animals.

## 2. Reservoirs of MPXV

**Figure 1:**
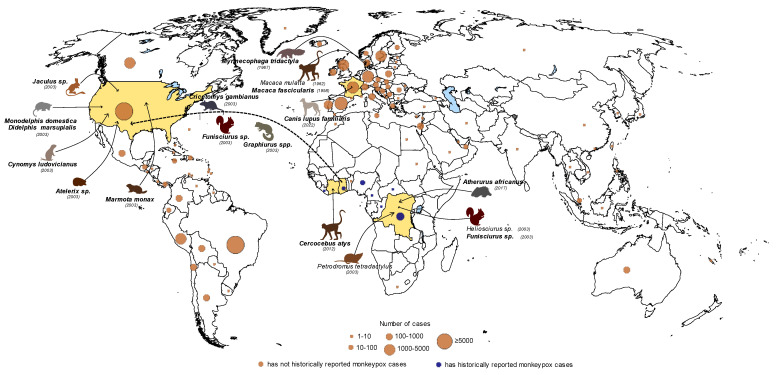
Confirmed cases of monkeypox in humans (orange and dark blue circles) and animals represented by countries. Countries that reported monkeypox infection in animals by PCR or viral isolation (bold italic species name) and serological method (italic species name) are marked with yellow. Dashed line indicates shipment of animals from Africa to the United States. The figure was based on continuously updated data available at the website of Centers for Disease Control and Prevention (data reported until 17 October, 2022) (https://www.cdc.gov/poxvirus/monkeypox/response/2022/world-map.html Accessed on 17 October 2022).

Since the first isolation of MPXV from captive cynomolgus monkeys (*Macaca fascicularis*), shipped from Asia to Europe in 1958, MPXV was thought to have originated from Asian primates [16]. Human cases were recognized and linked to monkeypox in Africa in 1970 during the smallpox eradication program that shed new light on the geographic origin of MPXV [17]. After the cessation of smallpox vaccination, 155 human MPXV cases were reported from West and Central Africa and the vast majority of cases occurred in small villages in the tropical rainforest, suggesting that indigenous African animals might be the reservoir hosts. During surveillance studies, including the detection of orthopoxvirus-specific antibodies by using virus neutralization and hemagglutination inhibition assays, a number of Central and West African, forest- and savannah-dwelling NHP species have been suspected as being potential hosts of the virus, such as *Cercopithecus* (*C. aethiops*, *C. ascanius*, *C. diana*, *C. mona*, *C. nicitans*, *C. petaurista*), *Colobus* (*C. badius*, *C. polykomos*) and *Allenopithecus* (*A. nigroviridis*) species [18,19,20]. In addition, pox-like disease and MPXV antibodies have been detected in the sera of squirrels (*Funisciurus*, *Heliosciurus*) and other rodents depending on the geographical area where MPXV infection occurred [21]. Furthermore, MPXV has been isolated from the skin and internal organs of a Thomas’s rope squirrel (*Funisciurus anerythrus*) captured in the Democratic Republic of the Congo (DRC) in 1985 [19,21,22,23,24]. Case reports suggested that several animal species could be susceptible to MPXV infection (e.g., giant anteater, short-tailed opossum, southern opossum, woodchuck, jerboa, African hedgehog) [25,26]. Nevertheless, attempts to isolate infectious MPXV from wild animals were less successful; an additional strain was obtained later, in 2012, from a sooty mangabey (*Cercocebus atys*) that died in a national park in the Ivory Coast (Figure 1) [27]. The cynomolgus monkey, the Thomas’s rope squirrel and the sooty mangabey, the hosts of the isolated MPXVs, showed skin lesions resembling poxvirus symptoms [21,22,27].

When screening programs have been carried out to estimate MPXV prevalence in humans and animals in DRC, orthopoxvirus-neutralizing antibodies were detected in the sera of squirrels (*Funisciurus anerythrus*, *F. congicus*, *Heliosciurus rufobrachium*), Gambian rats (*Cricetomys emini*), an elephant shrew (*Petrodromus tetradactylus*) and a domestic pig (*Sus scrofa*) [28]. In the same country, a specimen of a rufous-nosed rat (*Oenomys hypoxanthus*) has also been tested positive with the serological method [29]. Exposure of rodents in the genera *Cricetomys*, *Funisciurus*, *Graphiurus*, *Heliosciurus* (*H. gambians*) and *Xerus* to MPXV infection has been confirmed serologically and by polymerase chain reaction (PCR) from tissue samples in Central and West African countries (Figure 1) [23,24].

Collectively, serological evidence, DNA detection and, in a few cases, virus isolation implied many animal species as potential MPXV reservoirs, but the role of these animals in the perpetuation of natural MPXV infection is hard to interpret. Surveillance of captive wild animals, animals in trade and those used in infection experiments pointed out that a number of mammals are susceptible to MPXV. The stress and the proximity of miscellaneous species facilitate the spread of viral infections including MPXV, thus animals in captivity (in a zoo, sanctuary, shipment, shops or exchange events) are at higher risk of the disease [19,30,31].

**Figure 2:**
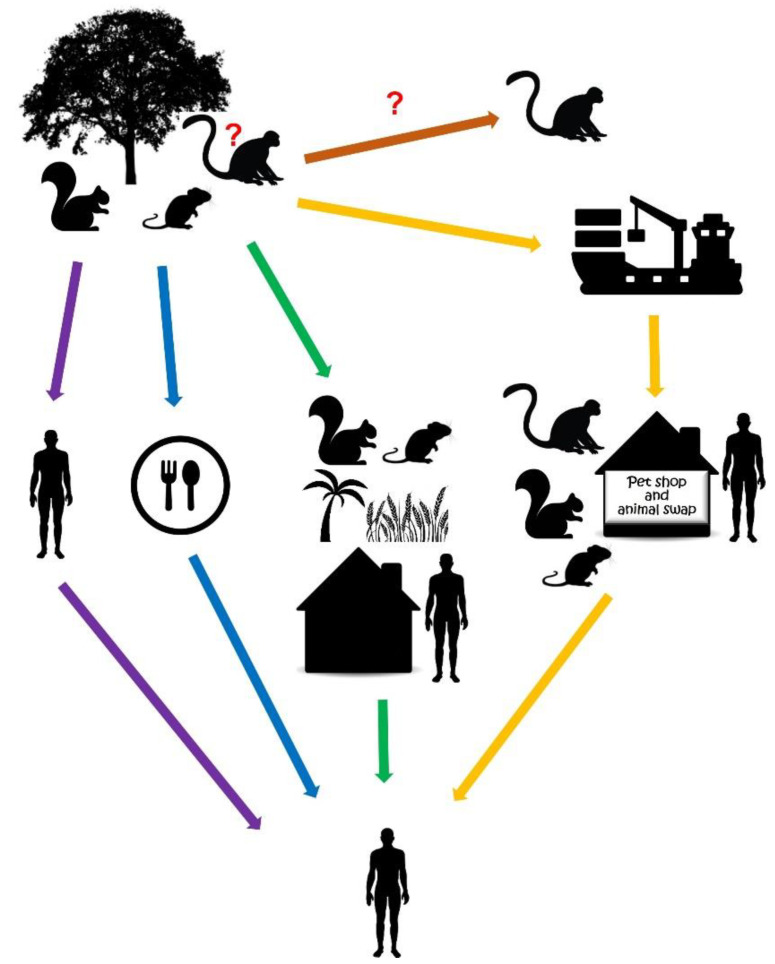
Potential transmission routes of monkeypox virus. Rodents have been suggested as natural hosts of MPXV that may transmit the virus to the human environment (green arrow). Close contact with the animals (green arrow) and with infected humans (purple arrow), consumption (blue arrow) and wild animal trade (yellow arrow) pose a risk to species spillover and zoonotic MPXV infection. Although non-human primates may be reservoirs of the virus, accidental infection of this group of animals is conceivable (orange arrow).

As suggested for other viruses, the disturbed and restricted habitat of wild animals may contribute to the spillover of the pathogen to humans that may, occasionally, lead to human-to-human transmission [22,32]. Besides close contact with animals, the consumption of wild animals has been taken into account as a source of human MPXV cases [23,28,33,34]. Although NHPs may be natural MPXV hosts, one may not ignore the possibility that these animals, similarly to humans, are accidentally infected by MPXV (Figure 2). At present, squirrels and other rodents are considered natural hosts, sustaining the circulation of MPXV in the enzootic African area (Figure 1). This group of animals may readily transfer the virus from the wilderness, primarily from the secondary forests, to the cultivated areas and human settlements (Figure 2) [19,22,32]. Taking all the evidence into consideration, the most likely reservoir hosts are *Funisciurus* squirrels [22,23].

Human cases, first identified in the USA in 2003, enabled the follow-up of an infection chain that could be tracked back to wild animal imports from Africa. Monkeypox has been registered with a febrile vesicular rash as the most often noted sign in the affected patients. Black-tailed prairie dogs (*Cynomys ludovicianus*), sold as pets, have been suspected as the source of MPXV in the index patient [31,35]. The prairie dogs may have been infected by rodents from an African shipment that included brush-tailed porcupines (genus *Atherurus*), Gambian giant rats (genus *Cricetomys*), rope squirrels (genus *Funisciurus*), dormice (genus *Graphiurus*), tree squirrels (genus *Heliosciurus*) and striped mice (genus *Hybomys*). Some of the succumbed animals, such as dormice, a Gambian giant rat and rope squirrels, tested positive for MPXV (Figure 1) [30,31]. Although the original host(s) could not be identified, a number of interspecies spillover events have been uncovered in this outbreak. The wide range of susceptible animals draws attention to the need for regulated animal transport and to the risk of close wild-animal contact.

## 3. Animal Models Used to Study Infection Biology

Supposed natural reservoirs and closely related mammals have been tested and preferably used for experimental MPXV infections to model the viral life cycle, cellular processes, disease development and immune response. These experiments clarified that infected animals variably react to MPXV infection, showing differences in disease development and mortality rates that depend on the mode of infection and the age of animals [36]. At present, those animals are used primarily in infection experiments that are sufficiently susceptible and permissive to MPXVs, adequately represent the human infection, and are readily available and maintained. These include the most often used laboratory and wild-caught mammals, such as the black-tailed prairie dog, the African rope squirrel (*Funisciurus anerythrus*) and the Gambian pouched rat (*Cricetomys gambianus*). Macaque models of monkeypox have also been developed that are indispensable for testing the safety and efficacy of vaccines and therapeutic drugs.

### 3.1. Prairie Dog Model

MPXV-infected prairie dogs displayed an incubation period of approximately 10–13 days followed ~2 days later by generalized cutaneous lesions that make these animals an informative model to investigate the course of infection. Hutson et al. (2015) [7] challenged prairie dogs intranasally (IN) with CB or WA clade MPXV (equivalent amount of each virus, 8 × 10^3^ plaque-forming units (pfu)). Similar pathological changes attributable to viral infection were seen between the clades. Generally, dermal lesions characterized by epidermal vacuolation and inflammation along with varying lymphoid tissue necrosis were observed on day 9 post-infection (PI). Additionally, splenic necrosis was present in CB MPXV-infected animals. Notable pathological changes were seen in animals 12 days PI, including multifocal necrosis in the oral and pharyngeal mucosal epithelia, liver, nasal cavity, uterus, spleen, small intestines and lymph nodes. The highest viral load was measured on day 12 PI from tissue samples for MPXVs of both clades (Table 1). The highest level of the virus was observed in the nasal cavity of animals challenged with WA MPXV, while the liver yielded the highest viral load of CB MPXV, followed by the spleen, nasal cavity and cutaneous lesions. In addition, 74% of the tissues harvested from the CB-infected animals had higher peak viral loads compared to WA-infected animals. Data suggested that viral replication takes place at the primary site of infection followed by MPXV dissemination via the lymphatic spread. The disseminated cutaneous lesions were formed as a result of secondary viremia after lymph got into the venous blood flow. Consistent with a previous study [37], it was also found that CB MPXV-infected prairie dogs shed a higher concentration of infectious virus and have a slightly earlier viral kinetics timeline compared to WA MPXV, whereas viral shedding is maintained for a longer period of time in WA MPXV-infected prairie dogs. Infected animals transmit the virus to naïve animals, as demonstrated in the prairie dog-MPXV challenge system. Despite the transmission of the two MPXV clades being minimal via the respiratory route, CB MPXV showed a higher rate of transmission compared to WA MPXV [37]. Additional observations in this animal model revealed that viral invasion of cells might trigger the apoptotic response. Moreover, CB MPXV caused more prominent apoptosis within the spleen than WA MPXV. Further investigations into apoptotic pathways in response to MPXV infection may shed light on differences in pathogenicity between clades [7].

### 3.2. Squirrel Model

Wild-caught African rope squirrels were also investigated to determine tissue tropism and clinical signs attributable to MPXV infection. Rope squirrels were infected IN or intradermally (ID) with a recombinant MPXV strain from Central Africa (1x10^6^ pfu) engineered to express Firefly luciferase. Viral shedding has been monitored during the study by in vivo bioluminescent imaging, viral titration and real-time PCR methods. Primary skin lesions appeared on day 3 PI and lesions were typical for poxviral infection by day 6 PI observed on the skin and oral cavity of the ID-infected group. In the IN-infected group, oral lesions were more common on day 8 PI and most of the animals (3 out of 4) showed severe respiratory disease with increased respiratory rate and nasal discharge starting on day 9 PI. Classic poxviral lesions on the skin became visible in two animals between day 11 and 13 PI. Shedding of high amounts of the virus in both IN and ID groups indicated that transmission could occur independently of the route of infection. Viral shedding of animals was observed from day 3 PI (before the onset of clinical signs) to day 25 PI. The highest concentration of MPXV was measured in oral secretions, reaching a peak on day 8–11 PI and day 11–13 PI in the ID- and IN-infected groups, respectively (Table 1). The highest number of viral DNA was detected in lips, tongues and primary skin lesions; however, the latter was evident exclusively in the ID group. Similar to other species infected with MPXV, epidermal and pulmonary damage were observed in rope squirrels. In addition, renal lesions were also commonly seen. Interestingly, MPXV infection in rope squirrels was not characterized by hepatic or splenic impairment compared to other sciurids (ground squirrel, prairie dog) [40]. The study demonstrated that rope squirrels could serve as amplifying hosts for MPXV and shed a high amount of virus (up to 1.34 × 10^7^ pfu), supporting their potential role in the epidemiology of MPXV in Central Africa.

### 3.3. Gambian Pouched Rat Model

Gambian pouched rats developed cutaneous lesions following inoculation with MPXV (4 × 10^4^ pfu from either of the two clades) by the subdermal scarification route to mimic a bite/scratch from an infected animal. Systematic involvement was also observed after infection with both MPXV strains, which was evident by clinical signs and behavioral changes in animals such as weight loss and decreased activity. Primary lesions at the inoculation site were more severe than the disseminated secondary lesions that appeared on the trunk and on the fore and hind limbs. The highest amount of viable virus was obtained from swabs of the inoculation site on day 6 PI (10^8^ pfu/mL), followed by oral and nasal swabs of CB MPXV (10^7^ pfu/mL) and WA MPXV (10^5^ pfu/mL) with peak loads on days 9 and 12 PI (Table 1) [42]. In another study, IN- and ID-infected Gambian pouched rats shed up to 10^6^ pfu/mL of the virus with oral secretions that are proved to be an infectious dose for NHPs and other rodents as well. The ID route of infection was more pathogenic than the IN route. The most important finding of this study was that Gambian pouched rats can be infected with and shed MPXV regardless of clinical signs of disease as they did not become moribund. As both animals with or without clinical signs may shed the virus for several weeks, they can transmit MPXV to humans and other animals, making them a potential source of human MPXV infection [43].

### 3.4. Non-Human Primates

Early studies on MPXV pathogenesis showed that *M. fascicularis* challenged with 10^5^ pfu via IN, intramuscular and scarification routes develop similar disease progress. The generalized rash was observed in 11 out of 12 animals on day 7–11 PI, characterized by the typical papule, vesicle, pustule and scab appearance of MPXV infection over a period of 3–7 days. Lesions appeared on the soles of the feet, palms, buccal mucosa and soft palate. Intriguingly, *M. mulatta* seemed to be more resistant to MPXV after intramuscular inoculation. Although the disease course was similar compared to *M. fascicularis*, reduced severity and less pronounced lesions were detected [47]. *M. fascicularis* was further investigated by Saijo et al. with an IN challenge dose (10^6^ pfu) of WA and CB MPXV. Decreased body weight, loss of appetite, rhinorrhea, conjunctival discharge, diarrhea, irritability and skin rash were seen in WA-infected animals. Viral DNA was detected in the blood from day 4 PI and reached its peak by day 9 PI. After being infected with CB MPXV, one out of two investigated animals exhibited severe clinical signs, while the other had very mild clinical signs. Skin lesions were more severe compared with monkeys infected with WA MPXV and viral DNA levels were 10 times higher than those recorded for WA-infected animals [48].

Subcutaneous experimental infection with 10^6^ pfu of MPXV in the *M. fascicularis* model appeared more pathogenic than the IN route. The CB MPXV infection was fatal in three out of four animals. One out of three WA-infected animals died as well. The typical papulovesicular rash was observed on days 7–9 PI. CB-infected animals were characterized by higher numbers of lesions compared with WA-infected animals. Lymph nodes and thymus were affected in both study groups, and the most common symptoms were anorexia and diarrhea. The most significant difference was the appearance of lesions, as granulomatous inflammation was seen in the gastrointestinal tract organs, such as the stomach, small intestine and colon, in the CB-infected monkey but not in the WA-infected monkey. Unlike WA-infected animals, the lungs of animals challenged with the CB strain were entirely and diffusely affected by the infection [48]. Generally, CB MPXV was more virulent and affected respiratory, genito-urinary and gastrointestinal tract organs more severely than WA MPXV. Taking all the results into consideration, the respiratory challenge route is probably more suitable for modeling MPXV pathogenesis and testing vaccine efficacy.

## 4. Virulence Factors of MPXVs

The MPX virion is 200–250 nm in diameter and has a complex structure. Both the enveloped and membrane-coated extracellular viral particles and the matured, intracellular virions are infectious. The core comprises the nucleocapsid bound, ~197 kbp long linear dsDNA genome that contains ~190 predicted open reading frames (ORFs) [15,49,50]. The genome of poxviruses encodes enzymes allowing extranuclear replication, RNA expression and assembly of the virus; thus, these viruses are able to replicate in the cytoplasm of infected cells. Some enzymes, for example, initiators of replication, are structural components of mature virions [15]. Although intra- and interspecies recombination have been detected among orthopoxviruses, analyses of extant genome sequences did not reveal significant recombination events in MPXV [15,51].

The observed variation in virulence of WA and CB MPXVs can be explained, in part, by the strain-specific differences in coding potential [2,3,49,52,53,54,55]. The WA and CB strains differ in both the number and structure of encoded genes. Genomic comparison of WA and CB MPXVs revealed 171 and 173 functional genes, respectively, with 170 unique orthologs that shared 99.4% amino acid identity [49]. Based on a comparison of orthopoxvirus ORFs and cellular homologs, a set of genes have been identified as components that putatively interfere with the host cellular processes, including the immune response against the virus [49,50,56]. The immune evasion strategies of orthopoxviruses include the hiding of viral DNA, the prevention of receptor recognition by dsDNA binding proteins, the inhibition of interferon (IFN) expression and response, as well as interference with pre-apoptotic and pro-inflammatory processes. Although most data comes from studies on the vaccinia virus, experimental data generated by the usage of animal models and cultured cells are also available for MPXV [50,53,54,56,57,58,59,60,61].

The main cellular targets of MPXV, as revealed by infection of cynomolgus monkeys and rhesus macaques (*Macaca mulatta*), are monocytic cells and granulocytes/neutrophils that may promote viral dissemination and multi-organ involvement [60,61]. MPXV may induce the imbalance of immune cells and regulatory proteins by influencing cytokine production. In the rhesus model, the absolute number of NK cells increased in the blood and lymphoid tissues, while the migratory and functional activity of NK cells such as chemokine receptor expression and cytokine (IFN-γ and tumor necrosis factor alpha) secretion reduced [60]. Unlike other poxviruses, MPXV did not downregulate the expression of the MHC I molecule but inhibited T-cell activation in an MHC-independent way in cultured cells [58].

**Figure 3:**
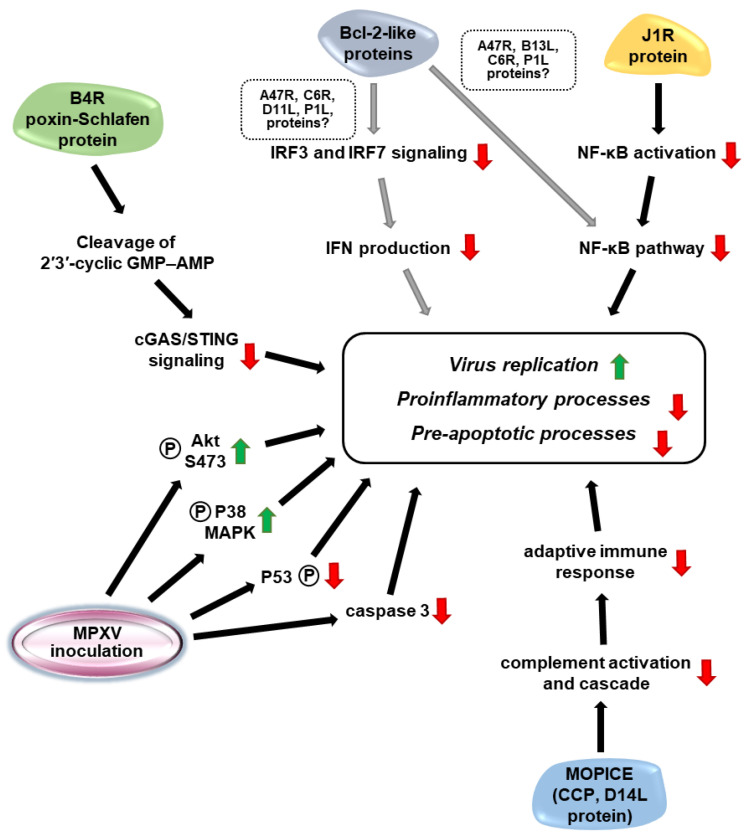
Schematic representation of cellular processes modulated by monkeypox virus proteins (green arrows, upregulation; red arrows, downregulation). Black arrows refer to experimentally verified result. Grey arrows show putative signaling concluded through mechanisms described for homologues of other orthopoxviruses.

To reveal differences in WA and CB MPXV infections, kinome arrays have been applied to inoculated monocytes. The results demonstrated an elevated level of phosphorylated Akt (protein kinase B) S473 in CB MPXV but not in WA MPXV infected cells. Inhibition of Akt S473 significantly decreased the viral yield of CB MPXV, displaying the impact of Akt-mediated signaling in the viral life cycle. Similarly, phosphorylation-based stimulation of the p38-MAPK (mitogen-activated protein kinase) pathway supported MPXV infection, with greater importance for the CB strain. Compared to the WA strain, CB MPXV had an increased anti-apoptotic effect connected to reduced p53 phosphorylation and lower caspase 3 activity, and promoted cell survival of monocytes (Figure 3). The triggering of apoptosis in both WA and CB MPXV-infected monocytic cells reduced the yield of infectious virions, with a 50× stronger effect on CB MPXV-infected cells, a finding that confirms the significance of anti-apoptotic processes in successful MPXV replication [54].

The cyclic GMP-AMP synthase (cGAS) receptors have a pivotal role in cytosolic viral DNA sensing that activates a cascade leading to IFN expression. Poxviruses encode poxin, a poxvirus immune nuclease that is encoded by the B2R gene in the vaccinia virus. This protein cleaves 2′3′-cyclic GMP–AMP (cGAMP), a messenger of the cGAS/STING (stimulator of IFN genes) signaling pathway that is responsible for IFN production. Poxins are conserved nucleases of orthopoxviruses, including MPXV, vaccinia virus and cowpox virus. In MPXV, the poxin (encoded by the B4R gene) is fused to the Schlafen-like protein domain that has been shown to share functional similarities (Figure 3) [57].

B-cell-lymphoma-2-like (Bcl-2-like) proteins of poxviruses have been identified as anti-apoptotic and anti-inflammatory molecules whose primary effect is the inhibition of NF-κB (Nuclear factor kappa B) signaling. NF-κB molecules are inactivated by IκB proteins and phosphorylation, ubiquitination and degradation of these inhibitors activate the NF-κB signaling. At the last, NF-κB transcription factor domain is translocated to the nucleus and transactivates the appropriate genes. Poxvirus proteins, including presumably MPXV proteins (A47R, B13L, C6R, P1L), interfere with NF-κB activation that may be responsible for the inhibition of pro-inflammatory and apoptotic processes. Furthermore, these proteins (A47R, C6R, D11L, P1L) inhibit the IFN regulatory factor 3 and 7-associated signaling, hampering IFN production (Figure 3). The anti-apoptotic state of a cell is obtained by caspase inhibitors, while inflammatory defense reactions are blocked by inhibition of pathways mediated by cytokines such as tumor necrosis factors, interleukin-1B and -18 [56]. Although these functions of the putative MPXV Bcl-2-like proteins have not been proven experimentally for MPXV, and even upregulation of apoptosis-inducing signaling has been detected in MPXV-infected *Macaca mulatta* kidney cells, homology among orthopoxviruses predicts similar functions in the anti-apoptotic processes during MPXV infection [62].

The J1R protein (ankryin/F-box containing protein) of MPXV and the ortholog G1R of other orthopoxviruses stabilize and inhibit the degradation of NF-κB subunit 1/p105, a precursor of NF-κB p50 subunit. The interaction prevents the formation of an active NF-κB p65/p50 complex and translocation of the NF-κB p65 transcription factor to the nucleus. As a result, the J1R (and G1R) protein restricts NF-κB mediated gene expression (Figure 3). Furthermore, the J1R (and G1R) protein interacts with S-phase kinase-associated protein 1, isoform b (SKP1A) of the SCF complexes that may impede ubiquitination and degradation of proteins, including that of the NF-κB inhibitor IκBα. However, the correlation between these two effects of J1R (and G1R) proteins needs to be clarified [59].

Monkeypox inhibitor of complement enzymes (MOPICE, D14L gene), a homologue of vaccinia and variola virus complement control proteins (CCPs, called VCP and SPICE for vaccinia and variola viruses, respectively), is a virulence factor of CB MPXVs, missing from the WA strains [49]. All of these CCPs perturb complement activation and cascade, thus inhibiting the molecular network and leading to virus neutralization (Figure 3) [49,52,53,63]. Black-tailed prairie dogs infected with MOPICE-lacking recombinant CB MPXV developed a less severe disease, while the viral quantities did not change significantly compared to the reference. Incorporation of CCP into a WA strain genome did not exacerbate extremely the virulence, but signs appeared earlier compared to the wild-type virus infections. The MOPICE may influence the cell tropism of the virus and may have an adverse effect on the adaptive immune response [53]. The MOPICE-lacking recombinant CB virus induced increased viral load and severe illness and perturbed adaptive immune responses in rhesus macaques [52].

At present, our knowledge is very limited about the structural and non-structural MPXV proteins and their function. A better understanding of the virulence factors could greatly assist in the production of effective target-specific vaccines and the revealing of potential therapeutic targets. The zoonotic properties of the virus and the available animal models are of high impact on the development of such formulations.

## 5. Animal Models Used in Vaccine Development

Despite smallpox being eradicated worldwide in the 1970s, vaccine research continued because of the concern that the variola virus (the causative agent of smallpox) could be used in biological warfare. NHPs helped to explore the course of orthopoxvirus infections, commonly using MPXV as a surrogate model of smallpox. MPXV infection causes similar symptoms in these animals as smallpox infection in humans, making them ideal models for testing candidate vaccines. MPXV infection of macaques provided valuable information for understanding disease pathology and the utility of existing smallpox vaccines against monkeypox as summarized by Cann et al. (2013) [64] and Parker and Buller (2013) [47]. Most recently, the marmoset (*Callithrix jacchus*) model has become a promising and inexpensive alternative to NHPs [65].

Although vaccines against smallpox that contain the vaccinia virus, a relatively benign orthopoxvirus, provide some cross-protection, there is currently no specific preventive tool against monkeypox. Cross-protection may be explained by the high similarity of vaccinia virus, MPXV and variola virus proteins. Variola and MPXV infections have very similar clinical manifestations but differ in their pathogenicity in humans. Smallpox is highly transmissible among humans by airborne droplets, contact with vesicle fluid and even with contaminated clothing, and has a very high mortality rate, causing death in almost one-third of the infected individuals [66]. Monkeypox may be less efficiently transmitted and has a lower mortality rate, with between 1 and 10% of outbreaks occurring mostly among young adults and children [6,67].

There are three generations of smallpox vaccines, of which the first generation is no longer licensed, and only second- or third-generation vaccines are recommended for vaccination (according to FDA updates on 24 September, 2019) (https://www.fda.gov/emergency-preparedness-and-response/mcm-issues/smallpox-preparedness-and-response-updates-fda#vaccines. Accessed on 24 September 2019). The first generation of vaccines contained live vaccinia virus and were produced by harvesting lymph from the skin of live animals after smallpox infection. In general, two strains were used for vaccination; the New York City Board of Health (NYCBH) strain, the freeze-dried form of which was marketed as Dryvax in the United States, and the Lister strain [68]. They were administered by scarification with a bifurcated needle, causing a typical pustular skin lesion on the site of vaccination [69]. The second generation of vaccines still contains live vaccinia viruses derived mostly from the NYCBH or Lister strains but harvested from the chorioallantonic membrane of chicken embryos or cell cultures [70]. ACAM2000, derived from the NYCBH strain grown on Vero cells, was approved in the USA in 2007, replacing Dryvax for smallpox vaccination and is still authorized for use today [71]. Although these vaccines are effective in protecting against disease and fatal infections, they can also cause severe adverse effects that should be considered [72].

Third-generation smallpox vaccines contain replicating or non-replicating attenuated vaccinia viruses and therefore have a better safety profile than first-generation vaccines. LC16m8 is a replicating attenuated, cell-cultured smallpox vaccine, developed and licensed in Japan in the 1970s. It is derived from the Lister strain and has lower virulence and replication competency because of the frameshift mutation in B5R, a major extracellular enveloped virion antigen [73]. Its safety and efficacy were tested against the following three poxviruses in different animal models: in mice against variola virus, in rabbits against rabbitpox virus and in NHPs against MPXV infections [74]. The efficacy of LC16m8 against monkeypox was tested in a cynomolgus monkey model and compared it with its parental strain, Lister. The side effects were milder for LC16m8 vaccination; only small skin lesions appeared at the vaccine take site with no satellite lesions. The immunized animals showed no monkeypox-associated symptoms after the challenge, whether they were infected IN or subcutaneously. MPXV viremia was not noted in the vaccinated IN groups, while decreased viral load was measured for the vaccinated, subcutaneous-inoculated animals. The naïve, non-vaccinated, challenged animals showed a higher viral load in all cases. Cytokine and antibody responses and histopathological lesions also affirmed these findings. In the vaccinated groups, vaccinia virus antigen-specific IgG became detectable two weeks postimmunization and a low level of cytokine response could be observed after the challenge. On the contrary, in the unvaccinated group, specific antibody levels were detectable only after the challenge, and IFN-γ and interleukin-6 levels increased when infected with MPXV. Furthermore, the internal organs of the monkeys in the unvaccinated group were affected by MPXV, while no lesions were detected in the vaccinated groups [75]. The LC16m8 vaccine was also used safely in immunocompromised (B- or T-cell deficient) cynomolgus monkeys, compared to Dryvax and caused only mild side effects, a skin lesion at the site of vaccination. The size of the lesion did not correlate with the number of B-cells in the blood, regardless of the vaccine used, and the same observation was found in the T-cell-depleted LC16m8 vaccinated group. On the contrary, in the Dryvax vaccinated group, the T-cell count inversely correlated with the lesion size, indicating that the immune system weakened by T-cell depletion is able to control the attenuated LC16m8 but not the nonattenuated Dryvax vaccine [76]. It has been also proved on a cynomolgus monkey model that even a single dose of LC16m8 vaccine can develop long-lasting protective immunity against MPXV infection. LC16m8 vaccinated NHPs were challenged at 6 and 12 months after vaccination and developed no monkeypox-associated symptoms [77].

Modified vaccinia virus Ankara (MVA) is a non-replicating attenuated vaccine strain, currently marketed under the names Imvamune, Imvanex and Jynneos, the latter two of which are licensed for use against smallpox and also monkeypox in the EU and in the USA, respectively (Imvanex and Jynneos were approved on 31 July 2013 and 24 September 2019, respectively) (https://www.ema.europa.eu/en/news/ema-recommends-approval-imvanex-prevention-monkeypox-disease; https://www.fda.gov/emergency-preparedness-and-response/mcm-issues/smallpox-preparedness-and-response-updates-fda#vaccines Accessed on 24 September 2019) [78]. The safety of MVA was tested in immunosuppressed cynomolgus monkeys, and although the presence of the MVA genome was detected by PCR in the animals, no replicative virus was isolated and they did not show symptoms related to the replication of MVA after vaccination [79]. Investigations into the efficacy of MVA against monkeypox in cynomolgus monkeys revealed similar humoral and cellular immune responses compared to Dryvax. No local or systemic adverse effects could be detected after vaccination with MVA; furthermore, the skin lesions caused by Dryvax vaccination healed more rapidly when Dryvax was added in combination with MVA. Binding and neutralizing antibody titers were nearly equal in vaccinated animals when immunized with Dryvax or MVA alone but reached higher levels when MVA priming was followed by either MVA or Dryvax. Despite the low level of viremia detected in the blood of the vaccinated animals, they remained healthy after intravenous challenge, except for a small number of mild skin lesions. Furthermore, antibody titers (both binding and neutralizing) were raised faster in the previously immunized monkeys than in unvaccinated animals [69]. Similarly, macaques challenged respiratory with sublethal and lethal doses of MPXV were also protected from lethal monkeypox infection by the MVA vaccine [80]. On the other hand, MVA did not protect immunodeficient rhesus macaques from a lethal monkeypox infection. Although their reactions after vaccination were comparable to those of healthy NHPs, simian immunodeficiency virus (SIV) infected macaques became severely ill after the challenge and were euthanized. While depletion of CD8+ T-cells did not affect the protection against MPXV infection, deficiencies in immunoglobulin production, such as failure of B-cell response and IgM-IgG isotype switching could result in lethal infection [81,82].

Recently, DNA and protein-based subunit vaccines have emerged alongside the whole virus vaccines. There are the following two forms of infectious orthopoxviruses: the intracellular mature virion (MV) and the extracellular enveloped virion (EV). The major form of poxviridae is the MV, which is present in the host cell and is released only with its disruption, while EV is released from the cell by budding and is responsible for cell-to-cell spread. The membrane protein composition of MV and EV differs significantly, as EV has an additional membrane layer that should be taken into consideration when designing subunit or DNA vaccines in order to ensure adequate antigenic coverage for both infectious forms of the virus [83]. The 4pox DNA vaccine contains two MV-specific genes (L1R and A27L) and two EV-specific genes (A33R and B5R). L1R and A27L are targets of MV-neutralizing antibodies, while antibodies to B5R neutralize EVs, and A33R is the target of complement-mediated cytolysis [84]. The efficacy of the 4pox vaccine against monkeypox was tested on a rhesus macaque model, and it developed effective protection against lethal challenges with MPXV. The animals were vaccinated with the 4pox vaccine one or two years prior to infection, and there were no detectable levels of binding antibodies prior to the challenge. So, a booster vaccination administered by a gene gun was performed, which elicited an immunological memory response. Monkeys vaccinated with the 4pox DNA vaccine were protected from lethal monkeypox and also from severe disease; they developed only very mild clinical and laboratory indications of monkeypox [84]. The *E. coli* heat-labile enterotoxin (LT) as an adjuvant was used to enhance the immune response to the 4pox vaccine in rhesus macaques: antibodies against A33, B5 and L1 were detectable already after the first vaccine, and their levels increased strongly after boost vaccination, whereas antibodies against A27 were only detected after the booster. Vaccination protected the NHPs from severe disease; furthermore, the monkeys vaccinated with 4pox/LT did not have detectable levels of infectious virus in their oral secretions, so the vaccine also prevented the shedding of the virus [85]. 

The multivalent smallpox vaccine developed by Hirao et al. (2011) [86] contained the following eight different targets: the MV antigens A27L, F9L, H3L and L1R; the EV antigens A33R, A56R and B5R; the core antigen A4L. Cynomolgus monkeys were immunized three times and, one month following the third immunization, were challenged with a lethal dose of MPXV. Vaccination developed a robust humoral (binding and neutralizing antibody) and cellular (CD4+ and CD8+ T-cell) immune response and protected NHPs from severe disease after lethal challenge [86]. Long-term protection was achieved with a recombinant vaccine integrated with IL-15 as follows: cynomolgus monkeys were challenged three years after a single dose of vaccination. At the vaccine take site, skin lesions could be observed, and robust neutralizing antibody and CD8+ T-cell levels were measured after vaccination. Humoral and cellular immune responses were monitored regularly in the meantime of vaccination and challenge, and prior to the challenge, no neutralizing antibody levels could be detected. Despite this, a rapid rise in serum anti-monkeypox antibody titers was observed in all vaccinated animals after the challenge, and vaccination protected NHPs against lethal monkeypox [87].

A subunit recombinant vaccine candidate was tested as well [88]. Rhesus macaques were immunized with the following different vaccines: the 4pox vaccine containing cDNA plasmid, administered intramuscularly or ID; or with recombinant proteins L1R, A33R, B5R and A27L expressed in *E. coli*, administered intramuscularly; or a cDNA plasmid vaccination boosted by protein vaccination. Animals immunized with DNA prime/protein boost had the highest binding and neutralizing antibody titers and developed only a mild disease after the challenge. Intramuscular cDNA vaccination could not protect NHPs from severe disease, while macaques immunized with subunit vaccines with or without DNA priming were protected from lethal monkeypox. The mild disease could be observed in these groups; however, animals immunized with a combination of DNA and proteins recovered more rapidly. The use of adjuvants in protein-based vaccines can also enhance immune responses [89]. Recombinant A33, B5, L1 and A27 proteins were produced with the use of baculoviruses and used in the vaccine with aluminum hydroxide alone or in combination with CpG as adjuvants. The vaccines did not cause adverse reactions in cynomolgus monkeys and induced a robust production of antibodies against MPXV. The protein vaccine containing both aluminum and CpG as adjuvants produced the highest titer of antibodies and also the clinical signs after the challenge were the mildest in this group [89].

## 6. Conclusions

Active disease surveillance, early diagnosis and outbreak data collection that help healthcare systems implement any public intervention measures are essential to control the spread of an MPXV that has the potential to cause an epidemic. Natural and experimental MPXV infections in wild rodents and non-human primates have generated important knowledge to improve appropriate case management and reveal some aspects of the MPXV transmission cycle. Results from animal models indicate that multiple rodent species may be involved in the maintenance and transmission of MPXV and revealed differences in the transmissibility of the two MPXV clades, as was observed in human cases as well. Prairie dogs, African rope squirrels and Gambian pouched rats are functional animal models for the study of MPXV infection since similar clinical signs that were evident in humans, including pox lesions and disease progression, were observed in MPXV-challenged animals. Interestingly, Gambian pouched rats seem to be less susceptible to clinical disease compared to rope squirrels and prairie dogs. The LD_50_ of MPXV in the respiratory route is estimated at 7.8 × 10^4^ pfu in cynomolgus macaques, while in the prairie dog model, the LD_50_ of MPXV in the IN route is estimated at 5.9 × 10^3^ pfu. Rope squirrels can shed viable viruses up to 1.34 × 10^7^ pfu/ml, highlighting the risk of lethal infection in susceptible animal hosts. Moreover, the viral shedding in all animal models was high (~ 10^6^–10^8^ pfu/mL), enabling the MPXV transmission to naïve animals within and between mammalian species and contaminating the environment through fecal and oral shedding. However, the respiratory transmission of MPXV appears to be less efficient than close or direct contact within all animal models. More recently, natural infection of domestic dogs has also been reported [90]. The finding that multiple host species may serve as a source of infection complicates the picture of monkeypox epidemiology and raises long-term challenges for future control and prevention efforts.

## Figures and Tables

**Table 1:** Representation of the most often applied animal models of MPXV infection and the associated experimental results.

Animal Model	Inoculation Route	Infection Dose	Clinical Signs	Gross Pathology	Viral Shedding (Viral Titer/mL)	References
prairie dog	intranasal	1 × 10^8^ pfu CB or WA strain	disseminated cutaneous lesions, inappetence, nasal discharge	lymphadenopathy, inflamed oviducts, hemorrhagic foci of adipose tissue and lungs	7.8 × 10^7^ pfu (WA MPXV) 2.3 × 10^8^ pfu (CB MPXV)	Hutson et al. 2015 [7]
	intranasal	6 × 10^3^ pfu WA	lesions, crusty noses, dehydration and inappetence	not examined	2 × 10^5^–1 × 10^6^ pfu	Hutson et al. 2013 [37]
	intranasal	high dose 5 × 10^3^ pfu CB	inappetence, dehydration, nasal congestion, labored breathing, facial edema, swollen paws	not examined extreme morbidity	2 × 10^7^–6 × 10^7^ pfu	
	intranasal	low dose 7 × 10^2^ pfu CB strain	skin lesions, inappetence, labored breathing	not examined	1.2 × 10^4^–7.8 × 10^4^ pfu	
	intranasal	10^4^ pfu WA strain	maculopapular skin lesions distended abdomen, diarrhea, ocular discharge, weight loss	subacute, necrotizing dermatitis, severe acute necrosis of lymphoid tissue and fibrinoid necrosis of blood vessels in the thymus and tonsil, multifocal lymphoplasmacytic interstitial pneumonia	5 × 10^5^–4 × 10^7^ pfu	Falendysz et al. 2014 [38]
	intranasal	4.3–5.9 × 10^4^ pfu WA strain	skin lesions, inappetence, mild nasal discharge	not examined	1.2 × 10^6^–1.7 × 10^9^ pfu	Weiner et al. 2019 [39]
rope squirrel	intranasal or intradermal	1 × 10^6^ pfu CB strain	ID and IN group: skin and oral lesions, nasal discharge, lethargy only in IN group: severe respiratory disease	not examined	up to 1.34 × 10^7^ pfu	Falendysz et al. 2017 [40]
ground squirrel	intraperitoneal or intranasal	10^5^ or 10^6^ pfu WA strain	lethargy	IP group: centrilobular hepatocytic degeneration or necrosis in the liver, moderate-to- marked necrosis of the spleen IN group: multifocal steatosis of the liver, diffuse hepatocytic necrosis, moderate-to- severe necrosis of the spleen	not examined	Tesh et al. 2004 [41]
Gambian pouched rat	scarification	4 × 10^4^ pfu WA or CB strain	skin and tongue lesions, lesions near eyes, lethargy, weight loss, hypopigmentation	not examined	inoculation site: 10^8^ pfu oral and nasal shedding: 10^5^ pfu (WA) and 10^7^ pfu (CB)	Hutson et al. 2015 [42]
	intradermal or intranasal	10^6^ pfu CB strain	ID group: weight loss, skin lesions, vesicles on the tongue, necrosis of the gingiva, lethargy IN group: no clinical signs	not examined	up to 1.85 × 10^6^ pfu	Falendysz et al. 2015 [43]
dormouse	intranasal	2 × 10^4^ CB strain	dehydration, conjunctivitis	upper gastrointestinal hemorrhage, hepatomegaly, lymphadenopathy, lymphoid necrosis in the submandibular lymph nodes, spleen and thymus, hepatocellular necrosis in the liver	~ 10^5^ pfu	Schultz et al. 2009 [44]
mouse (BALB/c and C57BL/6)	subcutaneous or intranasal	10^5^ pfu WA or CB strain	SC group (CB strain): edema at the site of inoculation, weight loss (only in BALB/c) IN group (CB strain): weight loss SC group (WA strain): slight edema at the site of inoculation IN group (WA strain): no clinical signs	not examined	not examined	Hutson et al. 2010 [45]
mouse (BALB/c)	intraperitoneal	10^5^ pfu WA or CB strain	rough coat, inappetence, decreased activity, multifocal lesions on the skin of the feet	severely necrotic ovary	not examined	Osorio et al. 2009 [46]

CB: Congo Basin, ID: intradermal, IN: intranasal, IP: intraperitoneal, pfu: plaque-forming unit, SC: subcutaneous, WA: West African.

## Data Availability

Not applicable.
